# Phosphorus Doping in Si Nanocrystals/SiO_2_ msultilayers and Light Emission with Wavelength compatible for Optical Telecommunication

**DOI:** 10.1038/srep22888

**Published:** 2016-03-09

**Authors:** Peng Lu, Weiwei Mu, Jun Xu, Xiaowei Zhang, Wenping Zhang, Wei Li, Ling Xu, Kunji Chen

**Affiliations:** 1 School of Electronic Science and Engineering, National Laboratory of Solid State Microstructures, Collaborative Innovation Center of Advanced Microstructures, Nanjing University, Nanjing, 210093, China

## Abstract

Doping in semiconductors is a fundamental issue for developing high performance devices. However, the doping behavior in Si nanocrystals (Si NCs) has not been fully understood so far. In the present work, P-doped Si NCs/SiO_2_ multilayers are fabricated. As revealed by XPS and ESR measurements, P dopants will preferentially passivate the surface states of Si NCs. Meanwhile, low temperature ESR spectra indicate that some P dopants are incorporated into Si NCs substitutionally and the incorporated P impurities increase with the P doping concentration or annealing temperature increasing. Furthermore, a kind of defect states will be generated with high doping concentration or annealing temperature due to the damage of Si crystalline lattice. More interestingly, the incorporated P dopants can generate deep levels in the ultra-small sized (~2 nm) Si NCs, which will cause a new subband light emission with the wavelength compatible with the requirement of the optical telecommunication. The studies of P-doped Si NCs/SiO_2_ multilayers suggest that P doping plays an important role in the electronic structures and optoelectronic characteristics of Si NCs.

Si nanocrystals (Si NCs) have been extensively studied in the past few years due to their potential applications in advanced nanoelectronic and optoelectronic devices, such as non-volatile memories^1^, light emitting devices^2^,^3^,^4^,^5^ and the next generation of solar cells^6^,^7^,^8^. So far, most studies have been focused on the unintentionally doped Si NCs materials, and only a few works have been concerned with the impurity doping in Si NCs though it is a fundamental issue to develop the high performance Si NCs-based devices. Several studies have revealed that phosphorus (P) and boron (B) doping in Si NCs is quite different from their bulk counterparts^9^,^10^. The initial theoretical simulation indicated that doping in quantum confined materials is quite difficult due to the large formation energy of n- and p-type impurities, which was known as the self-purification effect^9^. Recently, both the theoretical and experimental studies have suggested that the doping effect of P and B in Si NCs is strongly affected by the chemical environment^11^,^12^,^13^,^14^,^15^,^16^. For example, it was expected theoretically that P atoms tend to stay in the core while B atoms prefer to locate near the surface for the H-passivated Si NCs^11^. However, Pi *et al*. studied the H-passivated free-standing Si NCs and drew a conclusion that P atoms resides at or near the surface of the Si NCs while B is in the core region^14^. The further studies pointed out that the discrepancy may arise from the imperfectness or surface passivation ( H or O) of the Si NCs^17^. Meanwhile, a few works reported the role of doping in Si NCs on the electronic transport behaviors^18^,^19^. It was found that a strong increase of conductivity with doping of individual Si NCs can be attributed to the contribution of P donors and Si dangling bonds via spin-dependent hopping process.

On the other hand, light emission from Si NCs is also an interesting topic because it can be potentially applied in the Si-based monolithic optoelectronic integrations^20^,^21^,^22^. Though the indirect bandgap has excluded the bulk Si as a good choice for light emitting material, strong luminescence from the Si NCs has been reported owing to the enhanced radiative recombination probability of electron-hole pairs caused by the quantum confinement effect^23^,^24^. Pavesi *et al*. demonstrated that optical gain is possible in Si quantum dots, which is usually observed in the direct-bandgap compound semiconductor lasers^20^. More recently, a direct bandgap-like emission was reported by combining quantum confinement with surface engineering^25^. However, the quantum confinement effect will enlarge the bandgap of Si NCs compared to its bulk value (~1.1 eV), which causes the emission wavelength blueshift to the visible light region. Therefore, the present emission wavelength does not fully meet the requirements of the optical telecommunication in which the most suitable wavelength is in the near-infrared (NIR) region (1300 nm–1600 nm). In order to get light emission with the photon energy below the Si band gap, rare earth doped Si NCs were usually studied as a promising material for the optical source. For example, P. J. Reece *et al*. demonstrated that Si microcavities with erbium ion implanted can emit light at 1550 nm which is a good match for the optical telecommunication^26^. Recently, it was reported that the subband light emission can be obtained in impurity-doped Si NCs, which provided a new approach to get the Si-based light emitter with the suitable wavelength^27^,^28^. However, the further utilization of Si NCs in future optoelectronic devices needs a deep understanding of the doping behaviors and the role of dopants on the electronic structure and optical properties of Si NCs, which has not been fully investigated so far.

In the present work, phosphorus-doped (P-doped) Si NCs/SiO_2_ multilayers were prepared by annealing amorphous P-doped Si/SiO_2_ stacked structures and the doping behaviors of P atoms were systematically studied by controlling the P doping concentrations and annealing temperatures. It was found that the P atoms tend to passivate the surface states (such as dangling bonds) of Si NCs during the formation of Si NCs via annealing process. Meanwhile, the low temperature electron spin resonance (ESR) spectroscopy revealed that part of the P dopants are incorporated into the Si NCs at the substitutional sites, which results in the ESR signal originating from the conduction electrons. At the low P doping concentration, the hyperfine structure can be identified, indicating that only one P dopant resides in Si NCs on average. With increasing the P doping concentration, the hyperfine structure disappears, which suggests that more P dopants stay inside the Si NCs. For samples with high P doping concentration annealed at high temperature (1000 °C), the ESR signal related to the defects (E’ center) is found. Furthermore, a subband photoluminescence (PL) peak at ~1300 nm is detected in the Si NCs/SiO_2_ multilayers, which is originating from the radiative deep levels induced by the incorporated P dopants. The wavelength of the emission light meets the requirements of optical telecommunication, which makes P-doped Si NCs a potential material for the monolithic optoelectronic integrations.

## Results

### Formation of Si NCs after high temperature annealing

**Figure 1:**
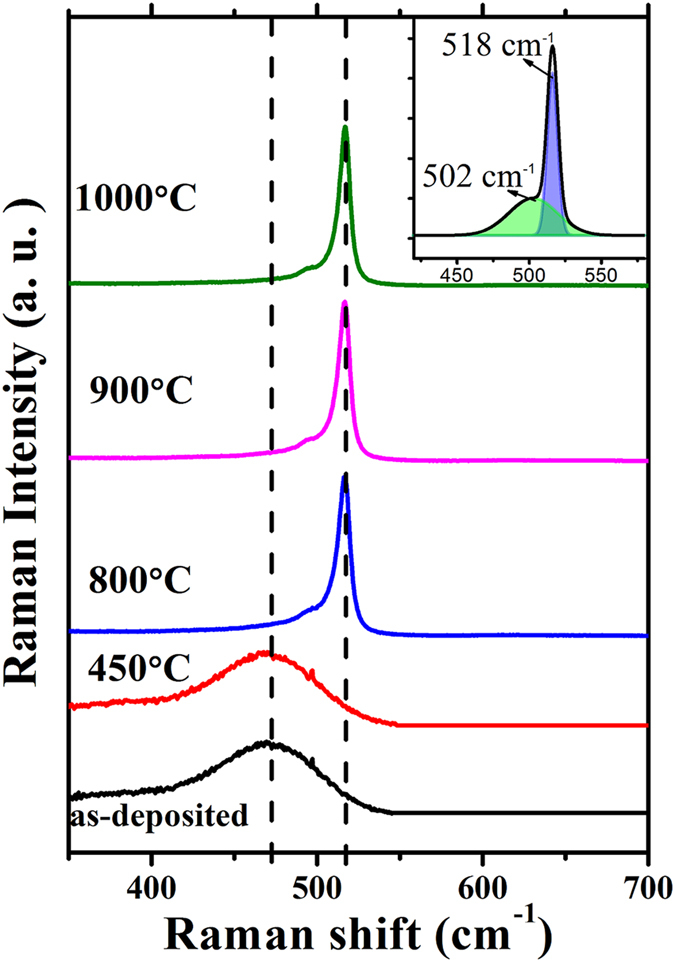
Raman spectra of P-doped Si NCs/SiO_2_ multilayers. The nominal P concentration is 2%. Inset is the decomposition of the Raman peak, with which the diameter of Si NCs is estimated to be ~6.6 nm.

Figure 1 shows the Raman spectra of the P-doped Si/SiO_2_ multilayers before and after annealing at various temperatures. The nominal doping concentration is 2% which is defined as the gas ratio of PH_3_ and SiH_4_. The thickness of the a-Si:H layer is controlled by the a-Si deposition time. When the deposition time is 15 s and 100 s, the layer thickness is estimated to be 2 nm and 13 nm, which is corresponding to the size of formed Si NCs of 2 and 6.6 nm, respectively^29^,^30^. For the as-deposited sample with the deposition time of 100 s, only a weak and broad Raman band centered at 480 cm^−1^ can be detected, which is associated with the TO mode of amorphous Si phase. After dehydrogenation at 450 °C, the samples still remain in the amorphous phase. When the annealing temperature is increased to 800 °C, a sharp peak near 520 cm^−1^ emerges, which indicates the formation of Si NCs. As shown in the inset of Fig. 1, The Raman spectra can be decomposed into two Gaussian peaks: an intermediate component located at ~502 cm^−1^ associated with bond dilation at grain boundaries^31^, and the crystalline component located at ~518 cm^−1^ due to the formation of Si NCs. Based on the phonon confinement model, we can estimate the mean size of Si NCs to be ~6.6 nm^32^.

### Location of P dopants in Si NCs/SiO_2_ multilayers

**Figure 2:**
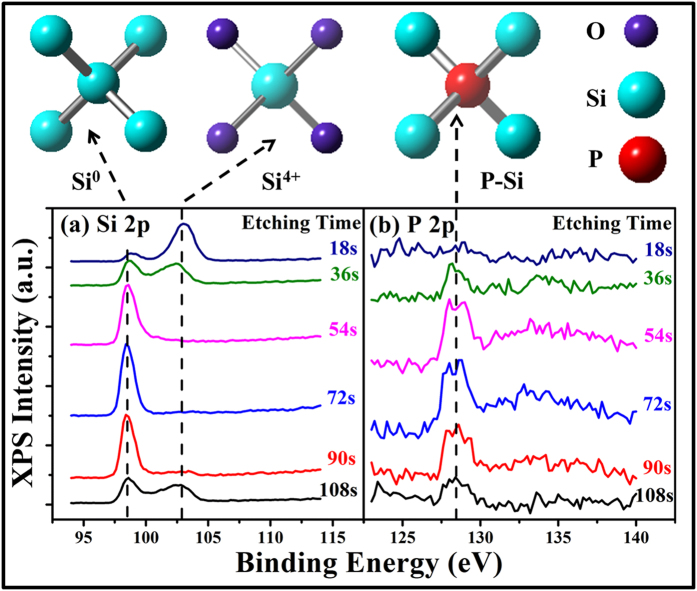
Depth-profile XPS spectra. The nominal P concentration is 2%. (**a**) Si 2p and (**b**) P 2p peaks for the P-doped Si NCs/SiO_2_ multilayers after 1000 °C annealing are detected. Insets are the schematic diagrams of Si-Si, Si-O and Si-P bonds.

Since we have confirmed the formation of Si NCs, it is necessary for us to study the location of P dopants. Fig. 2 shows the depth-profile XPS spectra of (a) Si 2p and (b) P 2p peaks for the 2% P-doped Si NCs/SiO_2_ multilayers after 1000 °C annealing. After 18 s etching (~5 nm), a strong peak at 102.9 eV is detected, which can be assigned to the Si^4+^ signals originating from the fully oxidized Si^33^, as shown in the inset of Fig. 2. It indicates that the detected position is in the SiO_2_ layer. At the same depth, no typical signals for the P-Si bond (128.4 eV) or P-O bond (134.5 eV) can be found in the P 2p spectrum, which implies that no P atoms exist in the SiO_2_ layer. When the etching time is increased to 36 s, the signal for Si^4+^ decreases and a peak associating with Si^0^ (~98.5 eV) appears. The coexistence of the two signals suggests that the detected position is near the Si/SiO_2_ interface. In the P 2p spectrum, a signal at 128.4 eV corresponding to the P-Si bond emerges, which implies that P atoms exist at the interface. When the etching time is further increased to 54 s (~15 nm), the peak for oxidized Si disappears completely and the peak for Si^0^ is strong and sharp, which suggests that the detected position moves into the Si layer. At the same time, we can find that the strong signal for P-Si bonds exists in the P 2p spectrum correspondingly, indicating that P atoms are distributed in the Si layer. The shapes of the Si 2p spectra and P 2p spectra don’t change until the etching time is 90 s (~25 nm), from which the thickness of the Si layer can be estimated to be ~15 nm. The estimated thickness agrees well with the pre-designed value controlled by the deposition time. When the etching time is increased to 108 s, the spectra for Si 2p level and P 2p level are similar to that at the depth of ~10 nm. The detected position moves to the Si/SiO_2_ interface of a new period. Besides, we find that the signal for P-O bonds (134.5 eV) cannot be seen at any depth. Based on the depth-profile XPS spectra, it can be identified that most of the P atoms locate in the Si layer and at the Si/SiO_2_ interface. Even after high temperature annealing, P atoms don’t tend to move into the SiO_2_ layer. In fact, the theoretical simulations also give the results that P atoms are not tending to stay in SiO_2_ due to a high formation energy^12^,^28^.

**Figure 3:**
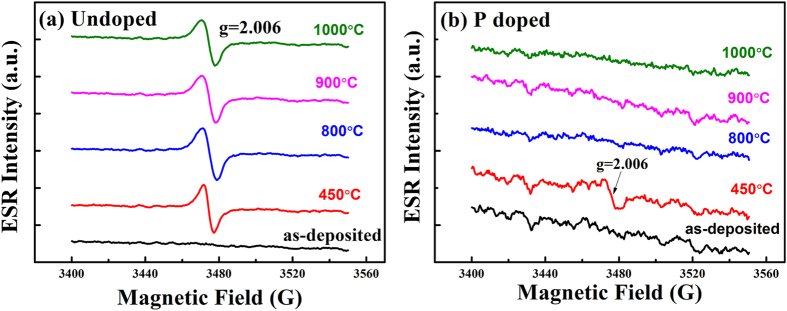
Room temperature ESR spectra. The room temperature X-band ESR spectra for (**a**) undoped and (**b**) 2% P-doped Si NCs/SiO_2_ multilayers. The annealing temperatures are varied to be 450 °C, 800 °C, 900 °C and 1000 °C.

In order to understand the doping behaviors of P impurities in Si NCs layers, room temperature X-band ESR spectra for (a) undoped and (b) 2% P-doped Si NCs/SiO_2_ multilayers with different annealing temperatures were measured, as shown in Fig. 3. For the undoped Si/SiO_2_ multilayers, no apparent signals can be seen in the as-deposited sample. After 450 °C dehydrogenation in N_2_ ambient, a signal with g = 2.006 is observed, which can be assigned to the Si dangling bond (DB) defects^34^,^35^. The Si DB defects are generated due to the effusion of H from the Si/SiO_2_ multilayers after 450 °C thermally annealing. When the annealing temperature is increased to 800 °C or higher, the signal for Si DB defects still exists, and the integrated intensity, which is proportional to the density of the Si DB defects, is enhanced (not shown here). This is because Si NCs are formed in the Si layer after high temperature annealing, and a large amount of Si DB defects are generated at the Si NCs/SiO_2_ interface. However, the ESR behavior for the P-doped sample is quite different from the undoped one. In Fig. 3(b), the Si DB defects emerge after the dehydrogenation process, which is similar to the undoped ones, but the defects disappear completely when the annealing temperature reaches 800 °C. This can be ascribed to the passivation effect of P atoms. After high temperature annealing, the Si NCs are formed and P atoms diffuse to the Si NCs/SiO_2_ interface to passivate the Si DB defects. In our case, the XPS spectra reveal that P atoms exist at the Si/SiO_2_ interface, and the ESR spectra can demonstrate the passivation effect of P atoms to the Si DB defects at the interface.

**Figure 4:**
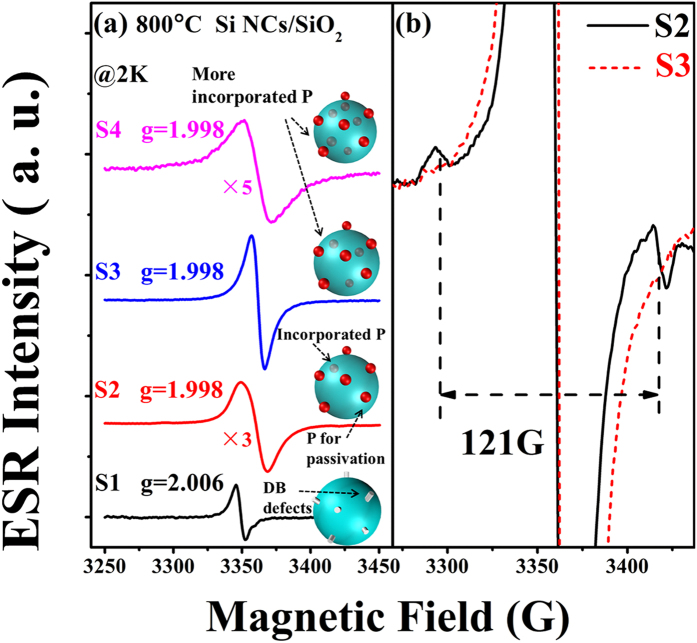
Low temperature ESR spectra. (**a**) X-band ESR spectra for Si NCs/SiO_2_ multilayers after 800 °C annealing obtained at 2 K. (**b**) HFS of S2 obtained at 20K. The ESR spectrum of S3 obtained at 20K is also provided for comparison. Insets are the schematic diagram of the P atoms in the Si NCs.

It is interesting to identify whether there is any P impurity inside the Si NCs. However, the room temperature ESR spectra for the P-doped Si/SiO_2_ multilayers are too noisy owing to the strong spin-lattice interaction under the high temperature. Low temperature ESR measurements can provide more detailed information about the electronic structures of the impurity-doped Si NCs materials^36^,^37^,^38^. Therefore, we measured the low temperature X-band ESR spectra for samples doped with various P doping concentrations and compared them with that of the undoped sample. Fig. 4(a) shows the X-band ESR spectra for 800 °C annealed Si/SiO_2_ multilayers obtained at 2K with the center field of 3360 G. The samples with the PH_3_ flow rate of 0, 0.3, 1 and 10 sccm, corresponding to the nominal P concentrations of 0, 0.06%, 0.2% and 2%, are denoted as S1, S2, S3 and S4, respectively. The spectra are scaled by certain multiplication factors for better comparison. For S1 (undoped sample), an ESR signal with g = 2.006 due to the Si DB defects at the surface of the Si NCs can be detected, which has already been observed in the room temperature ESR spectra. After P atoms are introduced, the signal for Si DB defects disappears and another signal with g = 1.998 emerges instead. This signal is assigned to the conduction electrons (CEs) in Si NCs^35^. These CEs are provided by the activated P atoms locating at the substitutional sites in the Si NCs. Besides, a hyperfine structure (HFS) can be seen in the spectrum for S2. To show the HFS more clearly, we choose the best observable spectrum which is obtained at 20K, as shown in Fig. 4(b). The ESR spectrum of S3 obtained at 20K is also shown for comparison. In this figure, two broad bands are located at about 3296G and 3417G on both shoulders of the CE signal for S2. However, no HFS can be seen and only the smooth CE signal remains in the spectrum of S3. This HFS arises from the interaction between the donor electron spin and the P nucleus spin^35^. When there is only one P atom in a Si NC, a donor electron will locate at the P nucleus and interact with the nucleus spin under low temperature, which is leading to the split of a single resonance line into a line doublet. If more than one P atom exists in a single Si NC, the HFS will disappear. In our case, the HFS can only be detected in S2, for which the doping concentration is comparably low. When the concentration is increased, the HFS cannot be detected any more.

**Figure 5:**
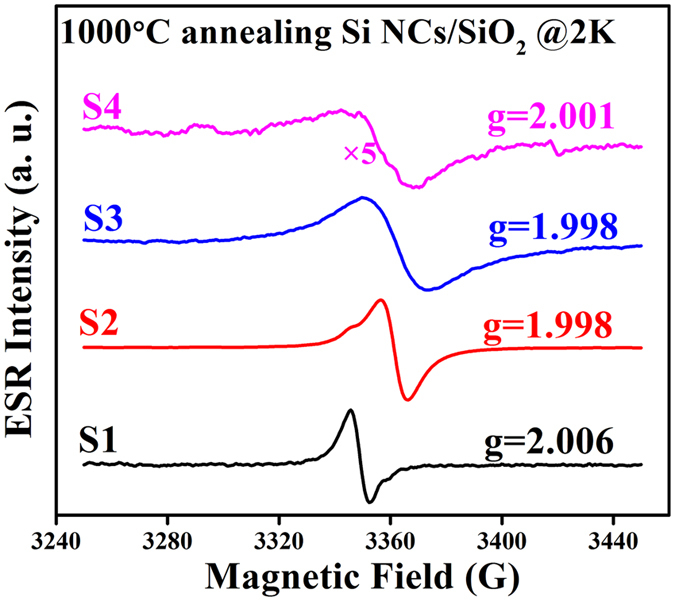
Low temperature ESR spectra after 1000 °C annealing. The X-band ESR spectra of Si NCs/SiO_2_ multilayers after 1000 °C annealing are detected at 2 K.

In order to understand more clearly about the doping behavior depending on the doping concentrations as well as annealing temperatures, the ESR spectra of S1-S4 after 1000 °C annealing were also obtained. Fig. 5 shows the X-band ESR spectra of Si/SiO_2_ multilayers obtained at 2 K with various P doping concentrations after 1000 °C annealing. The ESR signals are also scaled for better comparison. For S1, the ESR signal with g = 2.006 due to Si DB defects remains. When P atoms are introduced into Si NCs, the signal with g = 1.998 due to conduction electrons is observed, but the HFS cannot be found. It is because more P atoms move into the Si NCs after 1000 °C annealing. When the P concentration is further increased, different ESR behaviors are observed in the samples. On one hand, although the g value remains 1.998 for the CEs, the linewidth of ESR spectra for S2 and S3 is broadened. On the other hand, the ESR spectrum for S4 is much noisier, and the g value shifts to 2.001. In previous reports, the signal with g = 2.001 was attributed to the E’ center defects in the amorphous SiO_2_^39^,^40^. In our samples, the defects are attributed to the damage of Si crystalline lattice caused by the large amount of intensively moving P atoms. The detailed difference of the ESR signals under high annealing temperature will be discussed below.

### Structural characterization of ultra-small sized Si NCs/SiO_2_ multilayers

**Figure 6:**
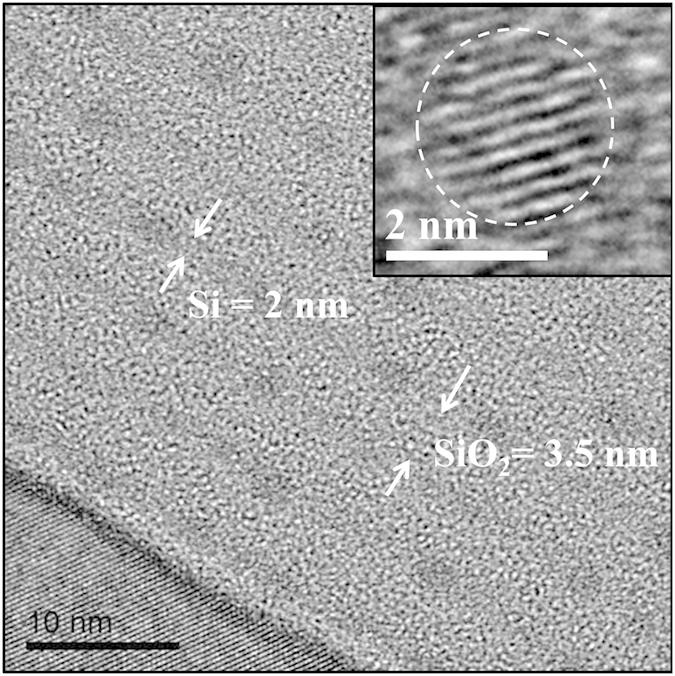
Cross-sectional TEM image. The average thickness of Si and SiO_2_ layers is 2 nm and 3.5 nm, respectively. Inset is the magnified image of a single Si NC. The diameter of Si NCs is about 2.0 nm, which is consistent with the Si layer.

It was theoretically expected that a radiative deep level will be induced in the bandgap by P impurities for the ultra-small sized Si NCs^41^. To better understand the role of P impurities in Si NCs, we fabricated the P-doped Si NCs/SiO_2_ multilayers with the ultra-small size. Fig. 6 shows the cross-sectional TEM image of the Si NCs/SiO_2_ multilayers after 800 °C annealing. Inset is the magnified image of a single Si NC. In this figure, both the Si and SiO_2_ layers are formed after the layer by layer deposition process. The layer thickness of Si and SiO_2_ is about 2 nm and 3.5 nm, respectively. Meanwhile, we can see that the dot-shaped Si NCs are formed after the subsequent 800 °C annealing, and they are uniformly dispersed in the Si layer. As shown in the inset, the crystalline structure can be identified and the diameter of the NCs is about 2 nm, which is consistent with the thickness of the initial a-Si layer.

### Photoluminescence properties of ultra-small sized Si NCs/SiO_2_ multilayers

**Figure 7:**
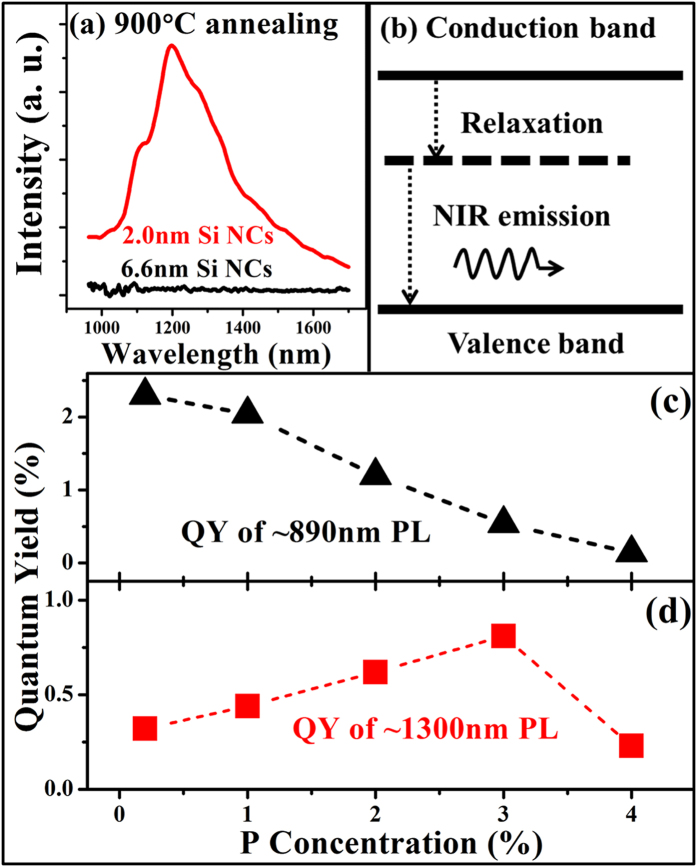
Room temperature spectra and the quantum yield of PL. (**a**) Room temperature PL spectra for the 2% P-doped Si NCs with the diameter of 2 nm and 6.6 nm, respectively. (**b**) Schematic diagram of NIR light emission mechanism in P-doped Si NCs. (**c**) Quantum yield (QY) of the PL centered at ~890 nm with various P doping concentrations. When the doping concentration is increasing from 0.2% to 4%, the QY is decreasing from 2.30% to 0.14%. (**d**) QY of ~1300 nm PL with various P doping concentrations. The QY is gradually increased from 0.32% to 0.81% with increasing the P doping concentrations from 0.2% to 3%. With further increasing the P concentration to 4%, the PL QY reduces to 0.23%.

Subsequently, the room temperature PL spectra of the 2% P-doped Si NCs/SiO_2_ multilayers with the dot sizes of 2 nm and 6.6 nm are measured, as shown in Fig. 7(a). It is found that the 2 nm sized P-doped Si NCs/SiO_2_ multilayers exhibit a broad subband near-infrared (NIR) emission band centered at ~1300 nm after 900 °C annealing. However, no PL signal can be detected in the 6.6 nm Si NCs. It should be noted that, we also prepared the 4 nm sized P-doped Si NCs/SiO_2_ multilayers and there is no PL signal in the NIR region, which further proves that the deep level can only be induced in ultra-small sized Si NCs. The light emitting mechanism is schematically shown in Fig. 7(b). The electrons are first excited to the conduction band, and subsequently relaxed to the deep level. Then a radiative recombination will occur between the deep level and the valence band to emit NIR light. Meanwhile, the absorbance spectra are also obtained in Supplementary Fig. 2. It is found that the optical absorption is significantly enhanced with the incident light wavelength below 500 nm and no apparent absorption can be found at around 1300 nm, which indicates that the electrons are excited from the valence band to the conduction band and no direct transition occurs between the P-induced deep level and the valence band.

**Figure 8:**
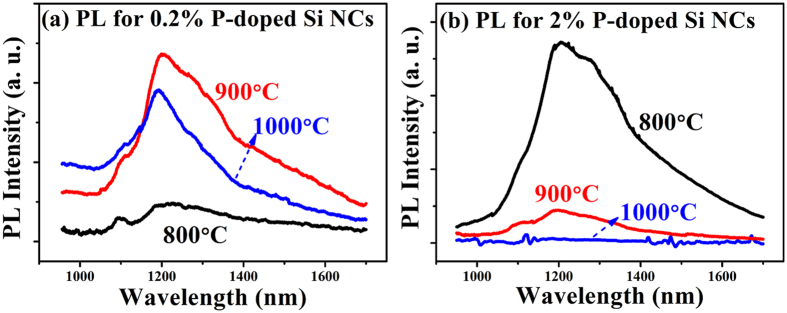
PL depending on concentrations and annealing temperatures. (**a**) Room temperature PL spectra for the 0.2% P-doped Si NCs/SiO_2_ multilayers. (**b**) PL spectra for the 2% P-doped Si NCs/SiO_2_ multilayers.

To further study the role of the P dopants in the subband light emission process, the PL behaviors of P-doped Si NCs/SiO_2_ with various doping concentrations and annealing temperatures were studied. Fig. 8 shows the room temperature PL spectra of the (a) 0.2% and (b) 2% P-doped Si NCs after high temperature annealing. In Fig. 8(a), only a weak PL peak at ~1300 nm can be detected for the 800 °C annealed sample because only a small part of Si NCs are incorporated with P dopants under the low P doping concentration. When the annealing temperature is increased to 900 °C, the PL intensity is enhanced. This is because more Si NCs are incorporated with P atoms under higher annealing temperature and more deep levels are generated. However, the PL intensity is slightly reduced when the annealing temperature is increased to 1000 °C. This reduction of the PL intensity can be assigned to the enhanced three-body Auger recombination process due to previous reports^42^,^43^ because more P atoms enter the Si NCs and provide electrons. Besides, the new defects generated by the P atoms under the high annealing temperature (1000 °C) are also responsible for the reduced PL intensity, which will be more apparent when the doping concentration is high. Fig. 8(b) shows the PL spectra of the 2% P-doped Si NCs/SiO_2_ multilayers. When the annealing temperature is 800 °C, the strong PL peak is detected. With increasing the annealing temperature, the PL intensity is decreasing rapidly and is completely quenched at 1000 °C. Here the Auger recombination is not the major reason for the PL reduction, and we should consider the defects revealed in the ESR signal for this sample. For the 1000 °C-annealed 2% P-doped Si NCs/SiO_2_ multilayers (S4), the defects with g = 2.001 are generated, which will act as electron traps and reduce the radiative recombination. When the P concentration or the annealing temperature is low, the effect of the defects is not apparent; when the P concentration or annealing temperature is high, a large number of defects will be generated, and the PL peak is quenched consequently.

## Discussion

By the Raman and depth-profile XPS measurements, it is demonstrated that P-doped Si NCs/SiO_2_ multilayers are obtained after annealing and P atoms are located in the Si layer or at the Si/SiO_2_ interface. Even after high temperature annealing, P atoms don’t tend to move into the SiO_2_ layer. Moreover, the room temperature ESR spectra for the undoped and P-doped Si NCs/SiO_2_ multilayers further reveal that P atoms will passivate the Si DB defects at the surface of the Si NCs. This result is also supported by other reports. Recently, Pi *et al*. showed that the P dopants tend to reside at or near the surface for H-passivated Si NCs^14^. Besides, Gnaser *et al*. claimed that a considerable amount of doped P atoms will stay at the interfacial layer of Si and SiO_2_ for the Si NCs embedded in SiO_2_ matrix^44^. Meanwhile, the theoretical simulation also suggested that P atoms prefer the surface sites for a lower formation energy^17^,^41^.

Although P atoms are demonstrated to stay at the surface sites of Si NCs, it is still unclear whether there is any P impurity in the inner side. In Fig. 4, the CE signal in the low temperature ESR spectra of P-doped Si NCs/SiO_2_ multilayers suggests that some P dopants are located at the substitutional sites inside the Si NCs. The HFS in the ESR spectra of S2 indicates that there is only one P atom existing in a single Si NC on average when the doping concentration is comparably low. For S3 and S4, the HFS cannot be detected, which indicates that more P dopants are incorporated with the concentration increasing. Moreover, the splitting of HFS given in Fig. 4(b) is about 121 G, which is higher than the value of the P-doped bulk Si (42G). This is because P donors are confined in a narrow space with a size comparable to the Bohr radius of P atoms (1.67 nm)^43^. With the splitting of the HFS, we can estimate the mean diameter of Si NCs to be about 6 nm^45^, which is close to the value calculated by the Raman spectra. It should be noted that, the temperature dependent ESR spectra of S2 and S3 are also obtained and the integrated intensity is studied (see Supplementary Fig. 1). The paramagnetism behaviors of the two samples also show that, with increasing the P doping concentration, more P atoms will be incorporated into the Si NCs. Hence, we can determine that P atoms are incorporated into the Si NCs and stay at the substitutional sites after passivating the Si DB defects at the surface. When the doping concentration is low, only one P atom enters a single Si NC; when the doping concentration is increased, more P atoms are incorporated, and only the CE signal exists. The schematic diagram is shown in the inset of Fig. 4(a). For S4, we notice that the ESR spectrum is rougher and broader than that of S2 and S3, and the peak to peak intensity is reduced though the g value is 1.998. The ESR intensity for S4 isn’t simply increasing with the doping concentration increasing, which indicates that different doping behavior of the P atoms arises when the doping concentration is high (2%).

The X-band ESR spectra of Si NCs/SiO_2_ multilayers after 1000 °C annealing are shown in Fig. 5. In this figure, we can find that although the g value remains 1.998 for the CEs, the linewidth of ESR spectra for S2 and S3 is broadened. Meanwhile, the ESR spectrum for S4 is noisier and broader, and the g value shifts to 2.001. The broadening for S2 and S3 is mainly due to the increased amount of conduction electrons. The integrated intensity of the ESR spectra can be calculated by the formula,


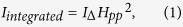


where *I* and _Δ_*H*_*pp*_ are peak-to-peak intensity and linewidth, respectively^37^. Meanwhile, the integrated intensity of the CE signal is proportional to the CE density. After 1000 °C annealing, more P atoms are incorporated into the Si NCs and activated, providing more electrons to the conduction band. Hence, the integrated intensity of the CE signal, which is proportional to the CE density, is increasing accordingly, resulting in the linewidth broadening. Besides, the increasing CE density shortens the distances between the electrons, leading to a stronger spin-spin interaction between CEs, which also contributes to the linewidth broadening.

For S4, a new ESR signal with g = 2.001 can be identified. In previous reports, this signal can be ascribed to the E’ center which is originating from the O vacancy defect in the amorphous SiO_2_^39^,^40^. In our samples, this similar signal can be assigned to the defects of Si crystal lattice caused by the moving P atoms. Under the high annealing temperature, the Si NCs are further oxidized and large amount of P atoms are intensively moving in or at the surface of the Si NCs. The P atoms will cause damage to the crystal lattice inside or at the surface of Si NCs, which will lead to the generation of O vacancy defects relating to the ESR signal with g = 2.001. These defects act as electron traps and hence make the CE signal disappear.

Based on our experimental results, we can summarize the doping behavior of P atoms in the Si NCs, as shown in the inset of Fig. 4(a). After 800 °C annealing, a-Si begins to crystallize and the Si NCs are formed. For the undoped Si NCs, the Si DB defects are generated at the surface due to the broken Si-H bonds after high temperature annealing. For the P doped Si NCs, the introduced P dopants will preferentially passivate the DB defects at the surface, and then be incorporated into the Si NCs. When the P doping concentration is low (0.06%), there is only one P dopant in a Si NC on average, resulting in the emergence of HFS. With the P concentration increasing, more P atoms enter the Si NCs and the HFS disappears. If the annealing temperature is increased to 1000 °C, more P atoms will be incorporated into the Si NCs. Moreover, when the P doping concentration or annealing temperature is high enough, the new defects will be generated due to the damage in the Si crystal lattice caused by the intensively moving P atoms.

Our observation is in good agreement with the theoretical expectation based on the density functional theory calculated by the formation energy of P impurities in the oxidized Si NCs^41^. The simulated results show that the introduced P atoms prefer passivating the Si DB defects at the interface of Si/SiO_2_ because the formation energy is the lowest. With all the Si DB defects passivated, P atoms can be incorporated into the Si NCs and stay at the sub-interface sites. They also reported that when a P atom stays at the substitutional site in the Si NCs with ultra-small sizes, a deep level will be generated in the bandgap. It is interesting that the P-induced deep level has a relatively high recombination rate and it will result in a subband emission, which is consistent with the previous reports that a subband light emission can be achieved in P-doped Si NCs/SiO_2_ multilayers^43^. Meanwhile, the radiative recombination rate will be obviously reduced with increasing the dot size.

Thus we fabricated the P-doped a-Si/SiO_2_ multilayers and obtained Si NCs with the size of ~2 nm after high temperature annealing. The room temperature PL spectra demonstrated that the radiative deep level only exists in the ultra-small sized Si NCs, which is consistent with the theoretical expectation. Meanwhile, the light emission mechanism is also shown in Fig. 7(b). In previous reports, similar subband light emission has been observed in P-doped Si NCs at the temperature as low as 5K by M. Fujii *et al*. In their work, an emission band centered at 0.9 eV was observed and it was attributed to the defect states at the interfaces between nano-crystalline Si and the matrix^27^. They also reported the PL signals from heavily P and B-codoped Si NCs. They found that, with increasing the P concentrations, the luminescence gradually shifted to the low energy. They explained the low-energy PL in terms of the transition between donor and acceptor states in nano-crystalline Si^46^.

Our present experimental results are quite different from the previous observations. The subband light emission in our case can be detected at room temperature. The quantum yield (QY) of the ~1300 nm light emission is calculated, as shown in Fig. 7(d). The QY is gradually increased from 0.32% to 0.81% with the P doping concentrations from 0.2% to 3%. When further increasing the P concentration to 4%, the PL QY reduces to 0.23%. The external QY of Si NCs was reported previously, which was only several percent^47^,^48^. For the NIR emission from Si NCs, it was reported to be 0.4% for a 1064 nm PL^48^. The measured QY of our samples is close to the values, and it is relatively higher than some other NIR emitters, such as PbSe quantum dots^49^. The dependence of the QY on doping concentrations can be understood as follows. Comparing with the Si NCs (~6.6 nm), it is more difficult for P dopants to enter the Si NCs with the ultra-small size (~2 nm). So it is natural that only a part of Si NCs are incorporated with P when the doping concentration is low. With increasing the P concentration, more Si NCs will have P atoms inside and more deep levels will be generated. Hence the PL QY will be increased accordingly. For the Si NCs with the highest P concentration (4%), more P dopants are incorporated into the Si NCs. The P dopants will provide more electrons, which will result in the apparent three-body Auger recombination process. Since the recombination time of the Auger process is much shorter than that of the radiative recombination^43^, the PL peak will be reduced accordingly. Another possible reason is that, if more P dopants are introduced into the ultra-small sized Si NCs, the new type of defects will be induced due to the damage of the crystalline lattice, which has been revealed by the ESR spectra for samples with high P concentration or high annealing temperatures, as discussed before. The new defects will also suppress the radiative recombination process. Another difference in our sample is that, the NIR light is accompanied with a short-wavelength light at ~890 nm which is usually observed in undoped Si NCs/SiO_2_ multilayers^28^. For P-doped Si NCs/SiO_2_ multilayers with low doping concentration annealed at 900 °C, both the PL band centered at ~890 nm and at 1300 nm can be detected. With increasing the P doping concentrations, the PL band at 890 nm is reduced rapidly and the PL band at 1300 nm is gradually increased as mentioned before. We also estimate the quantum yield (QY) of the emission band centered at ~890 nm. As shown in Fig. 7(c), the QY of the 0.2% P-doped sample is 2.30%, which is in good agreement with previous reports^50^,^51^. With increasing the P doping concentrations, the QY of the 890 nm PL is gradually reduced, which suggests that part of the electron-hole pairs recombine via other routes. Meanwhile, the QY of the 1300 nm PL is gradually increased with increasing the P doping concentrations from 0.2% to 3%. With further increasing the P concentration to 4%, the PL QY reduces. Considering the QY for the both emission bands, it is reasonable to imagine that the photo-generated electron-hole pairs can be recombined via the interface states and/or the P-related deep levels, and there is obviously a competitive relationship between the two kinds of recombination mechanism. Since the NIR PL intensity is enhanced with the P concentration increasing, we can determine that the radiative recombination preferentially occurs via P-related deep levels. In Fig. 8(a,b), the PL signals with various doping concentrations and annealing temperatures are compared. The PL spectra suggest that P atoms exhibit different doping behavior under high doping concentrations or annealing temperatures, and the defects generated by the moving P atoms will cause a strong reduction in the PL intensity. In order to further understand the generation of defects in heavily P doped samples annealed at high temperature and their influences on the luminescence behaviors, the further work, such as the ESR measurements for more P doped sample together with the DLTS studies, is needed to give us more information.

In conclusion, we have fabricated the a-Si/SiO_2_ multilayers in a conventional PECVD system and obtained the Si NCs/SiO_2_ multilayers by high temperature annealing. We give the basic information on the doping behaviors and doping effect of P impurities in Si NCs/SiO_2_ multilayers. By XPS measurements, we find that P atoms are distributed in the Si layer and at the Si/SiO_2_ interface. The ESR spectra reveal that P atoms will passivate the Si DB defects at the surface and then enter the Si NCs at the substitutional sites. With increasing the P doping concentration or annealing temperature, more P dopants can be incorporated into the Si NCs substitutionally. The introduced P dopants have strongly influenced the physical properties of Si NCs, especially for the ultra-small sized ones. For Si NCs with the ultra-small size (2 nm), P doping will induce a new deep energy level and result in a subband light emission centered at ~1300 nm. The PL intensity is gradually enhanced with increasing the P concentration. This is because more Si NCs are incorporated with P atoms to generate more impurity-related deep levels. However, when the annealing temperature is increased to 1000 °C or the P concentration is higher than 2%, the observed subband light emission is reduced and even quenched, which can be attributed to the generation of new defects states due to the damage of Si crystal structures as revealed by the ESR spectra. Our present results clearly suggest that the P impurities can be incorporated into Si NCs instead of merely staying at the interface states of Si NCs. The introduction of P atoms inside the ultra-small Si NCs can generate the impurity-related deep level to emit light with the wavelength compatible for the optical telecommunication, which opens a new route to the Si-based light source for monolithic optoelectronic integration.

## Methods

### Si NCs/SiO_2_ multilayers preparation

The undoped and P-doped hydrogenated amorphous Si/SiO_2_ stacked structures were fabricated on p-type mono-crystalline Si wafers (1–3 Ω/cm) and quartz substrates in a conventional plasma enhanced chemical vapor deposition (PECVD) system with a radio frequency of 13.56 MHz. The RF power was set as 50W and the substrate temperature was fixed at 250 °C during the deposition process. The a-Si:H layer was deposited by using SiH_4_ with a flow rate of 5 sccm. P doping was achieved by adding PH_3_ to SiH_4_ during the deposition process. The deposition time and flow rate of PH_3_ were varied to obtain the different layer thickness and doping concentrations. The deposition time for the 2.0 nm-thick Si layer and 13 nm-thick Si layer is 15 s and 100 s, respectively. The *in-situ* plasma oxidation was subsequently performed by using O_2_ for 90 s with a flow rate of 20 sccm. The deposition and oxidation process was alternatively repeated for several times to get multilayers. Subsequently, the as-deposited samples were first dehydrogenated at 450 °C and then annealed at various temperatures (800 °C, 900 °C, 1000 °C) for 1h under N_2_ ambient to form Si NCs.

### Raman scattering measurements

The Raman scattering measurements were performed to monitor the crystallization process by using a Jobin Yvon Horiba HR 800 micro-Raman system with an Ar+ laser (488 nm) as an excitation source. The flow rate of SiH_4_ and PH_3_ (1% diluted by H_2_) was 5 sccm and 10 sccm, corresponding to a nominal P concentration of 2%. The deposition time of the Si layer was 100 s, corresponding to the a-Si:H thickness of about 13 nm.

### XPS characterization

The X-ray photoelectron spectroscopy (XPS) measurements were performed with a PHI 5000 Versa Probe system and the composition signals of different depths under the surface were detected after Ar^+^ etching. The C 1 s line at 285 eV has been used as a reference to correct the charge shift of the binding energies. The etching rate is about 17 nm/min.

### ESR characterization

The room temperature X-band ESR spectra were obtained by using a ESR spectrometer of Bruker EMX 10/12 with the center field of 3480G. The low temperature X-band ESR spectra were obtained by using a liquid He cooled spectrometer of Bruker EMX 10/12+ with the center field of 3360G.

### PL characterization

The photoluminescence spectra in the range of 600–1000 nm were obtained by a HORIBA Jobin Yvon synapse CCD detector, while the spectra in the range of 900–1700 nm were obtained by using a liquid N_2_ cooled InGaAs detector. The excitation source is a 30 mW He-Cd laser working at 325 nm.

### Absorbance characterization

The absorbance spectra were obtained by using Shimadzu UV3600 (UV-vis-NIR) spectrometer in the range of 200–1600 nm.

### Quantum yield measurements

The quantum yield of the reference amorphous SiN film (PL peak at 470 nm) was measured and estimated by using a Xe lamp (Xenon short ARC) working at 325 nm as an exciting source and the photomultiplier detector (Hamamatsu 928 PMT, 300–850 nm) with an integrating sphere. The PL was collected by an optical fiber (Ocean optics) with the diameter of 1mm and detected by a fluoromax-4 system (Jobin Yvon). The QY for the reference was measured to be 6.6%. The QY of ~890 nm PL in our sample was calibrated by the reference by using a HORIBA Jobin Yvon synapse CCD detector in the range of 300–1000 nm. Since the QY of ~890 nm PL was obtained by comparing the sample with the reference, it was a relatively rough estimation. The QY of ~1300 nm PL was measured by a PL system (F920, Edinburgh Instruments) equipped with a 6-inch integration sphere. A calibrated Hamamatsu near-infrared photomultiplier tube was used to collect PL in the wavelength region of 900–1600  nm. The QY was calculated by the ratio of the number of emitted photons at 1300 nm to that of absorbed photons at 325 nm.

## Additional Information

**How to cite this article**: Lu, P. *et al*. Phosphorus Doping in Si Nanocrystals/SiO_2_ msultilayers and Light Emission with Wavelength compatible for Optical Telecommunication. *Sci. Rep.* **6**, 22888; doi: 10.1038/srep22888 (2016).

## Supplementary Material

Supplementary Information
