# Erratum: Phosphorus Doping in Si Nanocrystals/SiO_2_ Multilayers and Light Emission with Wavelength Compatible for Optical Telecommunication

**DOI:** 10.1038/srep33767

**Published:** 2016-09-21

**Authors:** Peng Lu, Weiwei Mu, Jun Xu, Xiaowei Zhang, Wenping Zhang, Wei Li, Ling Xu, Kunji Chen

Scientific Reports 6: Article number: 2288810.1038/srep22888; published online: 03 09 2016; updated: 09 21 2016

The original version of this Article contained an error in the title of the paper, where the word ‘‘multilayers” was incorrectly given as ‘‘msultilayers”. This has now been corrected in the PDF and HTML versions of the Article.

