# Supplementary material for: Phosphorus Doping in Si Nanocrystals/SiO2 msultilayers and Light Emission with Wavelength compatible for Optical Telecommunication
Source: Sci Rep. 2016 Mar 9;6:22888. doi: 10.1038/srep22888 (PMC4783703; doi:10.1038/srep22888)
Supplement: Supplementary Information [file srep22888-s1.doc]

**Phosphorus Doping in Si Nanocrystals/SiO_2_ multilayers and Light Emission with Wavelength compatible for Optical Telecommunication**

Peng Lu, Weiwei Mu, Jun Xu*, Xiaowei Zhang, Wenping Zhang, Wei Li, Ling Xu, Kunji Chen

**Supplementary information**


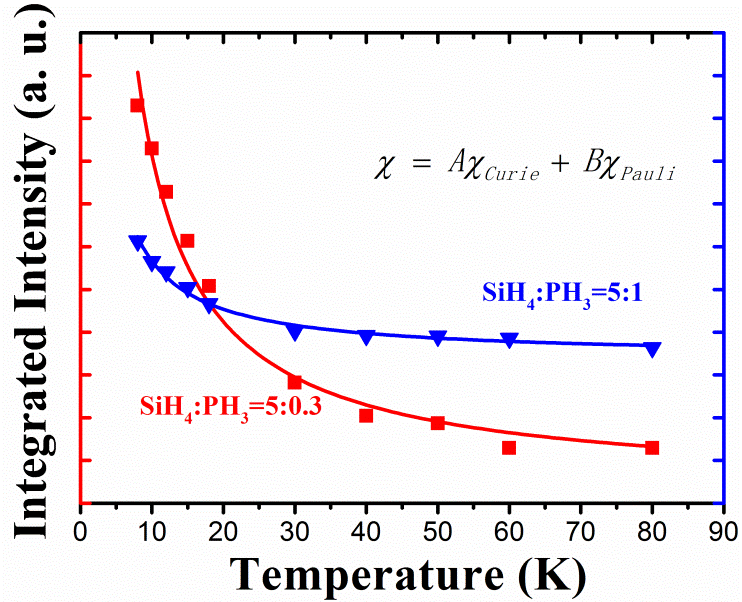


Figure 1 Temperature dependence of the integrated ESR intensity of Si NCs/SiO_2_ multilayers with various P doping concentrations.

The integrated ESR intensity of the conduction electron signal is proportional to the static susceptibility (χ). In P-doped bulk Si crystals, different doping levels lead to two kinds of paramagnetic mechanism[^1^](#_ENREF_1). If the P concentration is in the range of 10^16^ cm^-3^ to 10^18^ cm^-3^, the electrons will stay in their nondegenerate states, and the electron statistics are governed by the Maxwell-Boltzmann distribution. The susceptibility of the electrons obeys the Curie paramagnetism:

*χ_Curie_=(nμ_0_μ_B_^2^)/(kT),*

where *n* is the density of the paramagnetic centers (electrons), *μ_B_* the Bohr magneton, *μ_0_* the permeability of the vacuum, *k* the Boltzmann constant, and *T* the absolute temperature.

On the other hand, if the P concentration is in a high level, the paramagnetic susceptibility of the degenerated electrons at sufficiently low temperatures obeys the Pauli paramagnetism:

*χ_Pauli_=(nμ_0_μ_B_^2^)/E_F_*

*E_F_* is the Fermi energy. Obviously, the susceptibility is independent of the temperature.

In Fig. 1, the integrated intensity of the two samples is plotted as a function of the temperature. We fit the temperature dependence by the sum of Curie and Pauli paramagnetism. We find that in the 0.06% P-doped sample, the Curie part is about 77.6%. When the doping concentration was increased to 0.2%, the Curie paramagnetism behavior is strongly reduced and the proportion is only about 22.9%. The larger proportion of Pauli part can be ascribed to the increased amount of free electrons which are provided by activated P dopants at substitutional sites inside Si NCs. Hence, the temperature dependence of the paramagnetism behavior also proves that more P atoms will enter Si NCs with increasing the doping concentration.


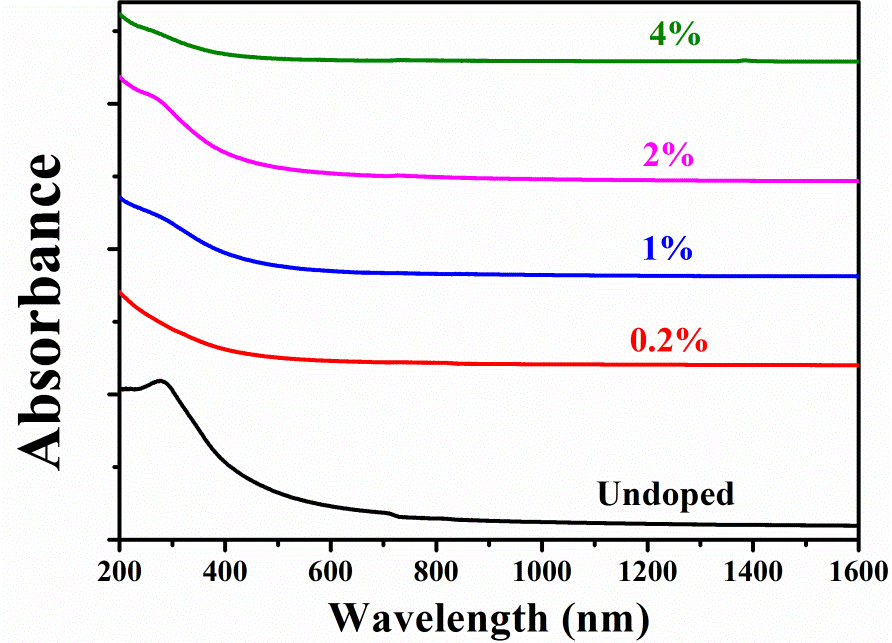


Figure 2 Absorbance spectra of undoped and P-doped Si NCs/SiO_2_ multilayers with various doping concentrations after 900℃ annealing.

Fig. 2 shows the absorbance spectra of undoped Si NCs/SiO_2_ multilayers and P-doped samples with the doping concentration of 0.2%, 1%, 2% and 4%. In this figure, we can find that the absorption is detected under the wavelength of 500nm, which indicates that the absorption is relating to the conduction to valence band transition since the optical bandgap of doped samples is about 1.6eV. Meanwhile, no absorption peak can be seen around 1300nm, which suggests that no direct electron transition occurs from the valence band to the P induced deep level. The absorbance spectra further prove that the electrons are first excited to the conduction band and then relaxed to the deep levels to emit the subband light.

**References:**

1. Muller, J., Finger, F., Carius, R. & Wagner, H. Electron spin resonance investigation of electronic states in hydrogenated microcrystalline silicon. *Phys Rev B* **60**, 11666-11677 (1999).
